# Supplementary material for: MetaRibo-Seq measures translation in microbiomes
Source: Nat Commun. 2020 Jun 29;11:3268. doi: 10.1038/s41467-020-17081-z (PMC7324362; doi:10.1038/s41467-020-17081-z)
Supplement: Supplementary file 10 — Supplementary Data 7 [file 41467_2020_17081_MOESM10_ESM.zip › File2/Confidence_VeryHigh_Taxonomy/136792_out.krona.html]

Javascript must be enabled to view this page.

members
magnitude
magnitudeUnassigned
count
unassigned
taxon
rank

136792\_out

6

2
superkingdom
6

6
1239
phylum

class
186801
6

6
186802
order

6
186803
family

1
46205
genus

species
1410616

SRS143780\_contig\_number\_11994
1

species
39491

SRS016018\_contig\_number\_13924SRS042628\_contig\_number\_38470SRS144135\_contig\_number\_35566SRS146812\_contig\_number\_60529
4

genus
572511
1

1

SRS097889\_contig\_number\_40021
33038
species
